# A Novel Murine Model to Study the Early Biological Events of Corticosteroid-Associated Osteonecrosis of the Femoral Head

**DOI:** 10.3390/bioengineering13010116

**Published:** 2026-01-20

**Authors:** Issei Shinohara, Yosuke Susuki, Simon Kwoon-Ho Chow, Pierre Cheung, Abraham S. Moses, Masatoshi Murayama, Mayu Morita, Tomohiro Uno, Qi Gao, Chao Ma, Takahiro Igei, Corinne Beinat, Stuart B. Goodman

**Affiliations:** 1 Department of Orthopaedic Surgery, Stanford University School of Medicine, Stanford, CA 94063, USA; isseihirai1027@gmail.com (I.S.); yo10bon.voyage@gmail.com (Y.S.); mayu5@stanford.edu (M.M.); tomo168@stanford.edu (T.U.);; 2 Department of Orthopaedic Surgery, Kobe University Graduate School of Medicine, Kobe 650-0017, Japan; 3 Department of Radiology, Stanford University School of Medicine, Stanford, CA 94305, USA; cheungp@stanford.edu (P.C.); mosesab@stanford.edu (A.S.M.); 4 Department of Bioengineering, Stanford University School of Medicine, Stanford, CA 94305, USA

**Keywords:** corticosteroid, osteonecrosis, oxidative stress, PET/CT, reactive oxygen species

## Abstract

This study establishes a murine model of corticosteroid-associated osteonecrosis of the femoral head (ONFH) using a sustained-release prednisolone pellet and evaluates mitochondrial stress using ^18^F-fluorodeoxyglucose positron emission tomography/computed tomography (PET/CT) and changes in key histologic markers of bone over a 6-week period. Sixteen 12-week-old Balb/C mice were divided into two groups: a prednisolone group (PRED) and a control group (SHAM). The PRED group received a subcutaneous 60-day sustained-release pellet containing 2.5 mg of prednisolone, while the SHAM group received placebo pellets. PET/CT imaging was performed at 1, 3, and 6 weeks. Bone mineral density (BMD) measurements, and histomorphological analyses for the number of empty lacunae, osteoblasts, osteoclasts, and NADPH oxidase (NOX) 2, a marker for oxidative stress, were conducted at 4 or 6 weeks. PET/CT imaging demonstrated increased uptake in the femoral head at 3 weeks in the PRED group. This was accompanied by increased numbers of empty lacunae and osteoclasts, increased oxidative stress, and decreased alkaline phosphatase staining at 4 weeks in the PRED group. We have successfully established and validated a small murine model of ONFH. The findings of this preclinical study suggest a critical timeline for potential interventions to mitigate the early adverse effects of continuous corticosteroid exposure on bone.

## 1. Introduction

Bone health can be significantly compromised in younger patients requiring long-term corticosteroid therapy for conditions such as rheumatoid arthritis and systemic lupus erythematosus (SLE), acute lymphoblastic leukemia (ALL), asthma, and other diseases [1,2,3]. Long-term corticosteroid use is linked to various bone-related conditions [4], including osteonecrosis (ON), osteoporosis, fractures, and growth suppression [5,6,7,8]. The incidence of corticosteroid-associated ON ranges from 1.6% to 17.6%, making it a common and debilitating side effect of anti-leukemic therapy, significantly impacting long-term quality of life [9]. The incidence of osteonecrosis of the femoral head (ONFH) is particularly high, affecting 35–67% of cases, and has a considerable impact on mobility [8,10]. The primary goal in managing corticosteroid-associated ONFH is early detection and preservation of the hip joint while considering the patient’s quality of life [11]. Therefore, understanding the pathogenesis and timeline for developing ONFH is crucial. Although the exact pathophysiology remains controversial, corticosteroids are known to disrupt the balance between oxidative and glycolytic metabolism, increasing reactive oxygen species (ROS) production and resulting in oxidative stress [12,13]. This increased oxidative stress is associated with prolonged inflammation, cell apoptosis, and impaired differentiation of mesenchymal stem cells (MSCs), leading to structural changes in the femoral head and secondary degenerative arthritis [14,15].

Oxidative stress from ROS often appears early after corticosteroid administration [16], which may help elucidate the early stages of ONFH. Although corticosteroid-associated ONFH adversely affects MSCs and subsequent bone formation, bone mineral density (using micro-CT [17]), and increases the number of empty lacunae and osteoclasts [18], the exact timeline for these changes has not been elucidated [19]. Positron emission tomography (PET) tracers have been developed to identify the role of ROS in biological processes. ROS-sensing radioactive tracers provide sensitive and quantifiable readouts for deep tissue imaging [20]. Sivapackiam et al. reported the use of PET/CT imaging for the noninvasive diagnosis of ROS-mediated acute lung injury and other chronic diseases [20]. PET imaging offers advantages such as high sensitivity, quantitative capability, minimal toxicity, and deep penetration [21]. ^18^F-fluorodeoxyglucose (^18^F-FDG), a non-metabolic analog of glucose, is FDA-approved as a PET imaging agent for use in oncology. This molecule is a marker for the Warburg effect, an inefficient method to produce ATP via aerobic glycolysis rather than oxidative phosphorylation [22]. This reflects a stressful local environment, characterized by mitochondrial damage or overload, probably due to an oxygen-poor environment. Furthermore, ^18^F-FDG has increased uptake during inflammatory processes, suggesting its potential as a diagnostic tool for early detection of osteonecrosis [23,24].

The purposes of this study are to establish and validate a clinically relevant murine model of ONFH, to examine the use of ^18^F-FDG PET/CT together with histomorphometric markers of oxidative stress and bone to identify the early events associated with corticosteroid-associated ONFH. This model could be used subsequently, to identify potential interventions to mitigate the adverse events associated this condition.

## 2. Materials and Methods

### 2.1. Animal Model and Prednisolone Pellet Selection

The experimental design was approved by the Institutional Animal Care and Use Committee (IACUC 34664), and institutional guidelines for the care and use of laboratory animals were followed in all aspects of this project. All mice were randomly assigned to either SHAM or the prednisolone treatment (PRED) group. We used sixteen 12-week-old male Balb/C mice (weighing 25 g to 29 g) in the first batch. Only male animals were used, to eliminate potential confounders due to hormonal difference between sexes. Sixty-day sustained-release pellets containing prednisolone (Innovative Research of America, Sarasota, FL, USA) were prepared for Balb/C mice [25], which is previously reported to demonstrate a short non-zero initial burst followed by a sustained slow-release [26]. Originally, we selected a 60-day sustained-release pellet containing 7.5 mg (5 mg/kg/day) of prednisolone based on a previous report [25]. However, mice treated with 7.5 mg prednisolone pellets became hyperactive on the day after surgery, followed by severe dehydration and weight loss, and the majority (6 out of 8 mice) were euthanized within 2 weeks. Therefore, we selected a significantly lower dose, 2.5 mg (1.6 mg/kg/day) of 60-day sustained-release prednisolone for this study. For these studies, 8 male mice in the PRED group and 8 male mice in the SHAM group were euthanized and evaluated at 4 and 6 weeks. These animals were used for the determination of body weight, bone mineral density (BMD), H&E staining for general histomorphological analysis and assessment of empty lacunae, and immunohistochemical evaluations of NADPH oxidase 2 (NOX2), a marker of reactive oxygen species, tartrate-resistant acid phosphatase (TRAP), and alkaline phosphatase (ALP). ^18^F-FDG PET/CT imaging was performed using a second batch of mice. The number of mice used per group and time points were as follows: SHAM (n = 4) and PRED (n = 5) at week 1; SHAM (n = 4) and PRED (n = 6) at week 3; and SHAM (n = 5) and PRED (n = 6) at week 6. Mice were housed in the Veterinary Service Center (VSC), Stanford with controlled temperature and 12 h light/dark cycles, provided ad libitum food and water, and body weight was monitored daily throughout the study.

### 2.2. Surgical Procedure and Evaluations

Inhalation anesthesia with isoflurane was used, and a small area above the scapula was shaved. A small 5 mm incision was made dorsally and the skin and subcutaneous were dissected. The pellet was inserted with tweezers into the pocket under the skin. The wound was sutured with 5–0 nylon thread and buprenorphine (3.25 mg/kg) was injected subcutaneously. After confirming the subcutaneous pellets with X-rays, the mice were returned to the cage and checked several times per week for weight, activity, and wound status. ^18^F-FDG PET/CT imaging was performed at 1, 3, and 6 weeks, and mice were euthanized at specific time points determined for each mouse. Mice were euthanized using carbon dioxide at a flow rate of 100% displacement/min for 10 min. Mice were then removed from the box and cervical dislocation was performed as a secondary method of euthanasia. Bone mineral density (BMD) was measured immediately after euthanasia, and the femurs were collected for histological evaluation.

### 2.3. ^18^F-FDG PET/CT Analysis

Each mouse was fasted overnight for at least 12 h prior to the start of the assay. Anesthesia was given through the inhalation of 4% isoflurane before injecting 100–150 µCi of ^18^F-FDG via the lateral tail vein using a 27 G butterfly catheter (BD Biosciences, Franklin Lakes, NJ, USA). The mice were then maintained under anesthesia at 2% isoflurane for 60 min before starting a 20 min PET acquisition with 4 mice placed simultaneously within the field of view (FOV). A standard CT with 80 kVp beam was acquired for attenuation correction and anatomical localization. PET images were reconstructed with the 3D-OSEM algorithm provided by the manufacturer. Both sequences were performed on a GNEXT PET/CT (Sofie, Culver City, CA, USA). The resulting data was analyzed through manual segmentation of the femoral head based on axial projections of the CT images using pmod 4.1 (Bruker, Kontich, Belgium). Data is presented as the percentage of injected dose per cubic centimeter within the voxel containing the highest activity (%ID_max_/cc). Scanning and evaluation of the mice was performed by a blinded researcher with no knowledge of the animal grouping.

### 2.4. X-Ray Analysis

Dual-energy X-ray Absorptiometry (DXA) was used to analyze whole-body areal bone mineral density (aBMD) and aBMD of the lumbar spine (L2–L5) [27]. BMD was measured after euthanasia at 4 or 6 weeks using a Faxitron UltraFocus DXA (Faxitron Bioptics, Tuscon, AZ, USA).

### 2.5. Preparation of Tissue Sections and Histological Analysis of Osteonecrosis Areas

After euthanasia, bilateral femurs were harvested from the mice. Femurs were fixed in 4% paraformaldehyde for 2 days and then demineralized with 0.5 M ethylenediaminetetraacetic acid (EDTA, Sigma-Aldrich, St. Louis, MO, USA) for 4 weeks. After demineralization, the femur was embedded in paraffin and 5-μm longitudinal sections were prepared, including the femoral head. Hematoxylin and eosin (H&E) staining was used in sections of the femoral head to assess the presence of empty lacunae. The sections were observed using a BZ-X 810 digital microscope (Keyence, Osaka, Japan) and six fields of view were randomly captured at 200× magnification. As in previous reports, the percentage of empty lacunae, a feature of dead cells in ONFH, was evaluated in the trabecular bone of the femoral head in each view [2]. For cell quantification, we used an automated validated cell-counting platform based on artificial intelligence technology developed by our group [28]. Briefly, the platform automatically classifies and counts normal osteocytes and empty lacunae using the object detection model You Only Look Once (YOLO) v8. The method has been validated by 3 independent observers. Comparisons were made between groups (n = 4 in each group) using the average of the percentage of empty lacunae in 6 pre-established fields of view for each mouse, similar to the method used in our previous studies in rabbits.

### 2.6. Immunohistochemical Analysis

Paraffin-fixed femur sections were also stained with tartrate-resistant acid phosphatase (TRAP), alkaline phosphatase (ALP), and NADPH oxidase (NOX)2. A TRAP histochemical staining kit (Sigma Aldrich, St Louis, MO, USA) was used for TRAP staining, and Fast Green (Sigma-Aldrich, St. Louis, MO, USA) was used as a counterstain. After staining, the cells were observed with BZ-X 810 (Keyence Corporation, Osaka, Japan), and TRAP-positive cells were defined as osteoclast-like cells, as in previous reports [29,30]. Six randomly selected fields of view of (0.1 mm^2^) using high microscopic magnification were used to quantify TRAP-positive cells and were evaluated by two blinded independent observers; the mean value of the observers was used. Osteoblast-like cells were identified using 1-Step™ NBT/BCIP Substrate Solution (Thermo Scientific, Rockford, IL, USA) for ALP staining [30]. Parameters related to bone formation were evaluated by the number of ALP-positive osteoblasts per trabecular perimeter (N.Oc/Tb.Pm) based on their anatomical location along trabecular bone surfaces and characteristic bone-lining morphology [31,32]. Quantification of ALP-positive osteoblast counts was performed by two evaluators using four random fields of view at high magnification.

For NOX2 staining, sections were processed with proteinase K (S3020, Dako Cytomation, Carpinteria, CA, USA) and endogenous peroxidase activity was blocked with 0.3% hydrogen peroxide treatment. Mouse monoclonal anti-rabbit NOX2 antibody (Proteintech, Rosemont, IL, USA) was used as the primary antibody at a 1:1000 dilution. The ImmPRESS^®^ HRP Horse Anti-Rabbit IgG Polymer Detection Kit (Vector Laboratories, Newark, CA, USA) was used for the secondary antibodies, and 3,3′-diaminobenzidine (DAB) solution (34, 002, Thermo Fisher Scientific) was used. Sections were then counterstained with hematoxylin. The percentage of staining in the cytoplasm and plasma membrane was observed at high magnification, and the percentage of brown-positive staining areas was quantified for each specimen using ImageJ version 1.54s (National Institute of Health, Bethesda, MD, USA). Positive NOX2 stained area was quantified in the trabecular region of the femoral head relative to the total bone area in the field of view. Quantification of staining ratio using ImageJ software in NOX2 staining was performed with reference to previous reports [30,33]. Briefly, ImageJ was used to split the RGB scale of the image to create an image consisting of red and blue, which are highly chromatic in brown. In each image, the degree of coloration is indicated by a uniform threshold value, and the staining area ratio is calculated. The color threshold for each stain was determined by consensus among the three investigators. Image analyses were performed by two blinded researchers independently, with no knowledge of the animal grouping (Figure S1 in Supplementary Materials).

### 2.7. Statistical Analysis

All data is expressed as mean and standard deviation. The Mann–Whitney U test was used for comparisons between two groups. Two-way ANOVA with Sidak’s multiple comparison test was used for comparisons among multiple groups. Statistical analysis was performed using Prism 9 (GraphPad Software, San Diego, CA, USA). Statistical significance was set at *p* < 0.05.

## 3. Results

### 3.1. Evaluation of Areas of ONFH by H&E Staining

**Figure 1:**
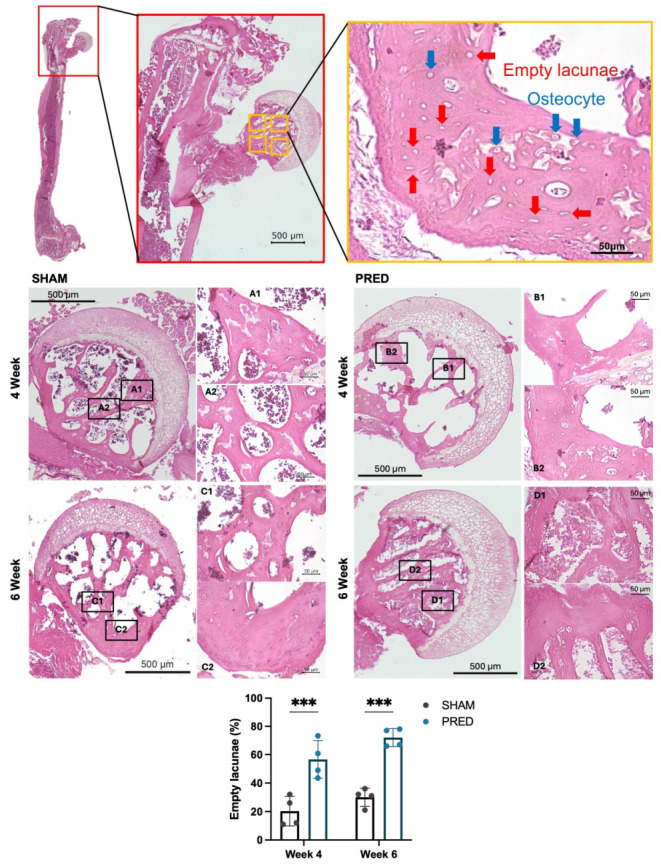
Representative images of hematoxylin and eosin (H&E) staining. Normal osteocytes (blue arrows) and empty lacunae (red arrows) were evaluated using high-power magnification. Note the dead marrow contents of the femoral head and delayed vascularization at the epiphyseal–metaphyseal junction in the group receiving corticosteroids. Cell quantification using an automated cell counting model (blue square: osteocytes, light blue square: empty lacunae). The percentage of empty lacunae was significantly higher in the PRED group than in the SHAM group at 4 and 6 weeks postoperatively (*** *p* < 0.001, n = 4 in each group). YOLO: You Only Look Once.

Figure 1 shows representative images at each endpoint (4 weeks and 6 weeks) for each group. The percentage of empty lacunae was significantly higher in the PRED group than in the SHAM group at both the 4- and 6-week time points (*p* < 0.001, Figure 1).

### 3.2. Evaluation of Osteoclastogenesis by TRAP-Positive Cells

**Figure 2:**
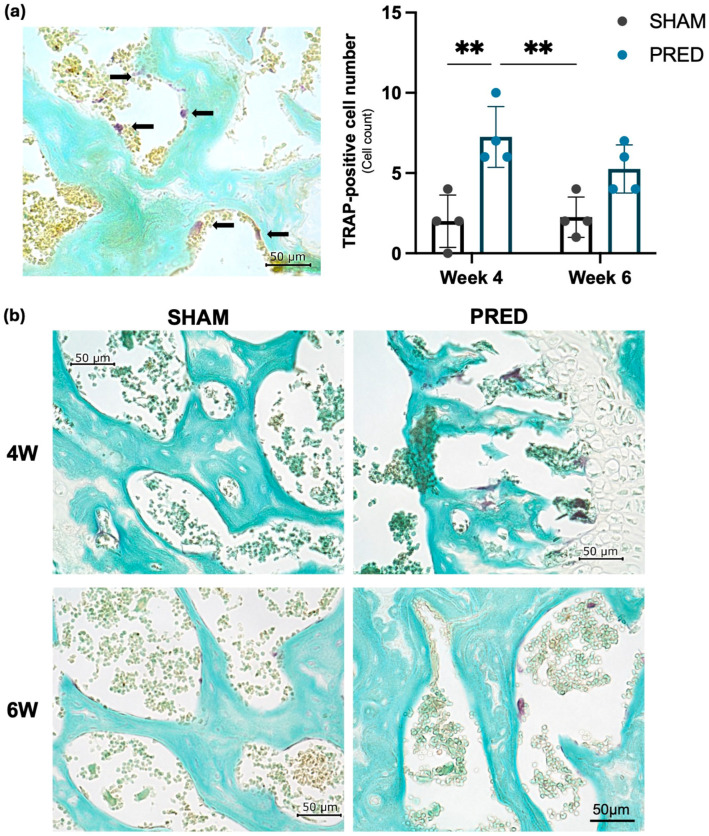
(**a**) Osteoclastogenesis was evaluated with tartrate-resistant acid phosphatase (TRAP)-positive cells indicated by black arrows. For quantification of TRAP-positive cells, six randomly selected fields of view were used from the high magnification (0.1 mm^2^) of the microscope. (**b**) Representative image of TRAP staining. TRAP-positive cells were significantly higher in the PRED group than in the SHAM group at 4 weeks postoperatively (** *p* > 0.01, n = 4 in each group).

The number of cells stained positive for TRAP was significantly higher in the PRED group than in the SHAM group at 4 weeks postoperatively (*p* = 0.03). At 6 weeks postoperatively, the number of cells stained positive for TRAP was not significantly different between the two groups. Representative images of TRAP staining are shown in Figure 2.

### 3.3. Evaluation of Osteoblasts by ALP-Positive Cells

**Figure 3:**
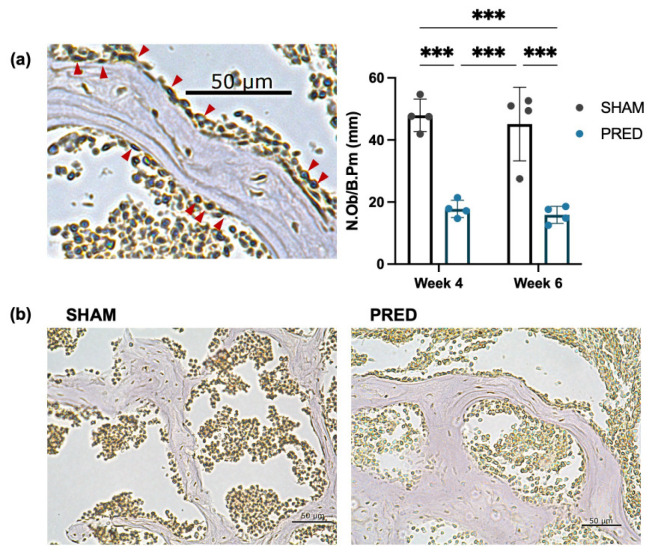
(**a**,**b**) Representative image of alkaline phosphatase (ALP) staining. The number of ALP-positive osteoblasts (red arrows) per trabecular bone was counted at high magnification of the microscope. The number of ALP-positive osteoblasts per trabecular perimeter was significantly higher in the SHAM group than in the PRED group at both weeks 4 and 6 (*** *p* < 0.001, n = 4 in each group).

The number of ALP-positive osteoblasts per trabecular perimeter was significantly higher in the SHAM group than in the PRED group at both weeks 4 and 6 (*p* < 0.01). Representative images of the SHAM and PRED groups are shown in Figure 3.

### 3.4. NOX2 Staining

**Figure 4:**
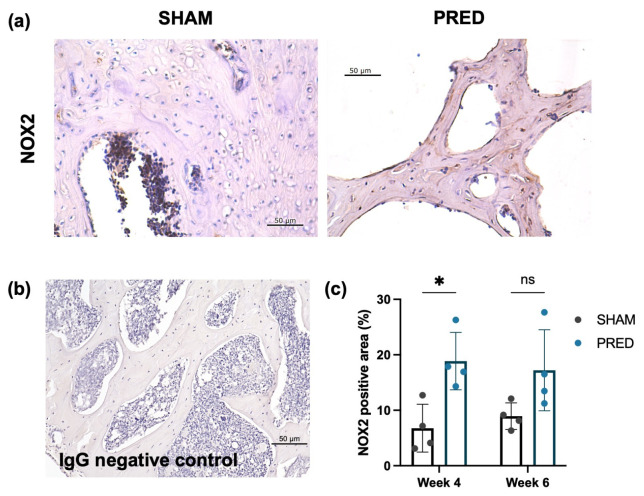
(**a**) Representative IHC images of Nox2 at the femoral head trabecular bones of the SHAM and PRED groups. (**b**) IgG-stained negative control images. (**c**) Significant difference detected at week 4 between the SHAM and PRED group. * *p* < 0.05, ns represents no significant difference.

NOX2 expression was higher in the PRED group than in the SHAM group at week 4 in the trabecular region of the femoral head, yet no significant difference was detected at week 6 (Figure 4).

### 3.5. ^18^F-FDG PET/CT Imaging

**Figure 5:**
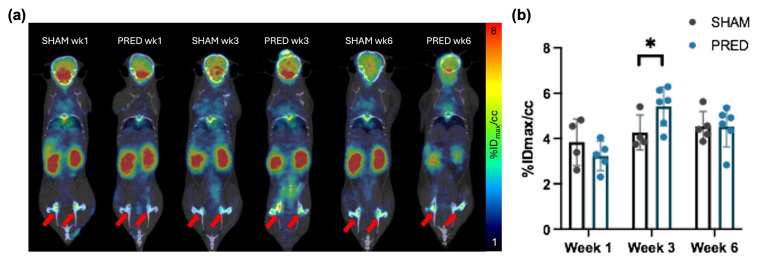
(**a**) Representative image of ^18^F-fluorodeoxyglucose PET/CT. The activity uptake within the ROI is expressed as the percentage of injected dose per cubic centimeter within the voxel containing the highest activity (%ID_max_/cc). Red arrows indicate bilateral femoral heads. (**b**) Uptake in the femoral head was higher in the PRED group than in the SHAM group at 3 weeks after treatment (* *p* = 0.04, n = 4–6 in each group).

^18^F-FDG uptake in the femoral head was significantly higher in the PRED group compared to the SHAM group at 3 weeks (*p* = 0.04). There were no significant differences between groups at weeks 1 and 6 (Figure 5).

### 3.6. Body Weight and BMD

**Figure 6:**
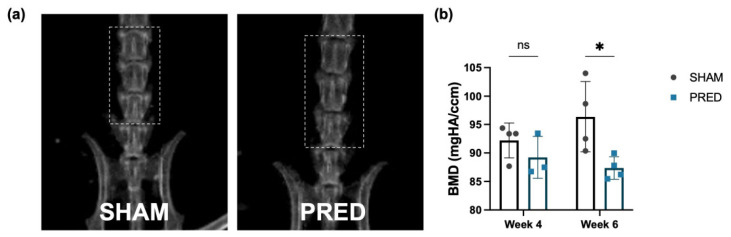
Bone mineral density at the lumbar spine at 4 and 6 weeks. (**a**) Representative images of bone densitometry of the 2 groups. (**b**) At 6 weeks, BMD of the spine in the PRED group was lower than in the SHAM group (* *p* = 0.04, n = 4 in each group, ns = no significant difference). PRED: prednisolone.

The body weight of mice in the SHAM group increased by an average of 9.3% from preoperative (26.8 ± 0.8 g) to 4 weeks postoperative (29.3 ± 0.8 g). In contrast, mice in the PRED group lost 3% of their body weight from preoperative (26.8 ± 1.5 g) to 4 weeks postoperative (25.8 ± 0.8 g). Whole-body and spine BMD at 4 and 6 weeks for each group are shown in Table 1, and representative images of radiographs for each group are shown in Figure 6a. BMD of the whole body and spine increased after 4 to 6 weeks in the SHAM group, while both decreased in the PRED group. After 6 weeks, BMD of the spine in the PRED group was significantly lower than in the SHAM group (Figure 6b, *p* = 0.04).

## 4. Discussion

The goal of this study was to identify the early events associated with corticosteroid-associated ONFH using a murine model and thereby determine potential interventions to mitigate the adverse events associated with this condition on bone.

Our results have shown that, by using a 2.5 mg sustained-release pellet prednisolone pellet subcutaneously, at 3–4 weeks, this model demonstrated increased uptake of ^18^F-fluorodeoxyglucose, a marker of mitochondrial stress reflective of oxidative glycolysis, rather than the normal metabolism of glucose via oxidative phosphorylation (also known as the Warburg effect). Furthermore, by 3–4 weeks, femora exposed to prednisolone showed increased numbers of empty lacunae (the hallmark of ONFH), increased osteoclastogenesis, increased oxidative stress, and decreased osteoblast activity, compared to femora exposed to sham pellets. This data reflects that prednisone is associated with dysregulated energy metabolism and oxidative stress, as well as abnormalities in bone homeostasis. Interestingly, at 6 weeks, some of these markers begin to normalize, including ^18^F-fluorodeoxyglucose uptake in the femoral head, as well as osteoclastogenesis, but not staining for empty lacunae, alkaline phosphatase, or NOX2. These findings probably represent attempts to re-establish normal local bone homeostasis despite ongoing corticosteroid delivery; the persistence of empty lacunae and decreased alkaline phosphatase expression probably reflects the residual effects of the prednisolone on osteoblast metabolism and function [34,35]. The development of osteonecrosis was consistent in 100% of the prednisolone-treated animals at 1.6 mg/kg/day, with 2–3 times increased empty lacunae compared to the sham controls.

We chose the 12-week-old mouse for these studies because, at this age, the mouse is physically and sexually mature and approximates a 20–30-year-old human, a common age for diagnosis and treatment of ONFH [36].

Animal models of corticosteroid-associated ONFH usually use one or more periodic injections of intramuscular methylprednisolone acetate injection in rabbits [37]. However, this protocol does not represent the scenario in humans in which corticosteroids are given daily and continuously for weeks or months. Furthermore, these models often employ additional injections of other factors such as lipopolysaccharide, etc., which do not mimic the events in humans and have a high rate of morbidity and mortality [34,38]. Our current model is clinically relevant and safe, simple, and has a 100% survivorship. In addition, there was a decrease in BMD after 6 weeks, which was consistent with models of corticosteroid-associated osteoporosis [25].

Although the exact biological mechanism of ONFH has not yet been fully elucidated, it has been suggested that this condition impairs osteoblast differentiation, increases osteoclast activity, and occludes capillaries that act as conduits to supply nutrients and cells to the bone repair unit [39,40]. Various vascular mechanisms have been proposed for the etiology of ischemia, including oxidative stress, thrombosis and coagulation, abnormal lipid metabolism, vasoconstriction, apoptosis, and necrosis [41,42]. As a result, in addition to decreased bone formation, ONFH results in ischemia and vascular occlusion due to the dysfunction of vascular endothelial cells, leading to apoptosis of bone cells. Therefore, the increased number of empty lacunae, a marker of cell necrosis in the bone trabeculae, is the benchmark to evaluate ON and to assess the efficacy of treatment [28,43]. In this study, the percentage of empty lacunae was significantly higher in the PRED group than in the SHAM group at 4 weeks, suggesting that ONFH was established in the PRED group by this time. TRAP staining also showed that osteoclast activity significantly increased in the PRED group at week 4. No significant difference was observed at 6 weeks, but osteoclasts have a lifespan of only a few weeks [44]. In contrast, ALP expression was significantly lower in the PRED group than in the SHAM group at both the 4- and 6-week time points. This decreased bone formation may be related to impaired differentiation of MSCs, and/or osteoblastic cell death caused by corticosteroids, which is associated with oxidative stress.

Long-term administration of corticosteroids causes oxidative stress by disrupting the physiological balance between oxidative and glycolytic energy metabolism and increasing ROS [13]. Oxidative stress may be closely related to the pathogenesis of ONFH, and indeed, studies using plasma samples from ONFH patients have indicated upregulated inflammation and abnormal energy metabolism due to oxidative stress [45]. Our group also reported that the addition of prednisolone to human MSCs in 3D culture caused oxidative stress and suppressed matrix calcification due to reduced cell proliferation rate and inflammation [46]. The expression of NOX2, the source of ROS production [47], was also increased by prednisolone treatment in the present study, supporting the relevance of oxidative stress in ONFH.

Early detection and therapeutic intervention are crucial in ONFH because of the significant impact of this condition on pain and function of the hip joint, and therefore quality of life [2]. In this study, we focused on the changes in glucose metabolism associated with oxidative stress and evaluated this marker using ^18^F-FDG PET/CT. Micro-PET provides real-time images of physiological parameters, may detect ON earlier than MRI scans, and could be more predictive of the progression and outcome of ON [48]. Specifically, PET/CT is presented as a research and translational imaging tool capable of detecting early metabolic and oxidative stress changes before structural collapse becomes evident. In fact, the utility of PET/CT in the diagnosis of osteonecrosis of the jaw has recently attracted attention [48]. Our study is the first to use PET/CT in an animal model to identify ONFH at an early stage. In our previous in vitro studies, the cell proliferative capacity of MSCs was significantly decreased by 1 week after prednisolone administration [46]. Mitochondrial dysfunction and inflammatory responses also occur during the acute phase, however, and ^18^F-FDG PET/CT was unable to distinguish a difference at 1 week. This finding may be due to the fact that, at 12 weeks, the physis in the mouse femoral head is characterized histologically by slightly disorganized and thin zones of resting and proliferating chondrocytes, without discernible zones of hypertrophy or provisional ossification. At week 13, the physis becomes resorbed, as seen with μCT, and replaced by cancellous bone. By 15 weeks of age, the physis is no longer observed on μCT or histologic analysis, indicating that complete resorption of the physis and epiphyseal fusion had occurred [49]. The key findings in this study are in accordance with this biological timeline. ON is simply not an epiphyseal phenomenon. Like ON in humans [50,51], our model using ^18^F-FDG PET/CT demonstrated concordant abnormalities in the metaphyseal area of the proximal femur at 3 weeks. It is also important to acknowledge that osteoblastic differentiation may involve a preferential shift to glycolytic pathways and the increased production of lactic acid [52,53]. However, the overall change in glucose uptake detected at the femoral head of the mice by PET/CT as a result of extended dosing of prednisolone may be due to other cell types, including osteoclasts and immune cells, other than osteoblasts alone [54]. This may also explain the transient changes in the ^18^F-FDG uptake during the 6-week period.

This study has some limitations. The series is small in sample size, but the results are compelling. A limited number of time points was explored. Sample sizes were determined a priori based on effect sizes reported in previous osteonecrosis studies using histological outcomes. The standard test for diagnosing ONFH in most clinical centers is magnetic resonance imaging (MRI), which is highly sensitive and specific for this condition [55]. However, CT is still used clinically by many centers, and is used during the staging system currently in use by The Association Research Circulation Osseous (ARCO) [56,57]. Furthermore, MRI is usually negative in the first few months after corticosteroid administration; in fact, in our clinical protocol, MRI is performed at 16 weeks after the initiation of corticosteroid treatment in young patients undergoing corticosteroid treatment for acute lymphoblastic leukemia. Other centers also perform sequential MRI studies beginning several months after corticosteroid initiation [58]. We are currently carrying out a study using the same model comparing the techniques of MRI and ^18^F-FDG PET/CT. A 12-week-old mouse does not perfectly replicate the complexity of a young adult human patient. However, this age corresponds to skeletal maturity in mice and approximates early adulthood in humans, which is a clinically relevant period for corticosteroid-associated ONFH. The strength of this model lies in its ability to define early biological events and therapeutic windows, rather than to directly predict clinical outcomes.

## 5. Conclusions

**Figure 7:**
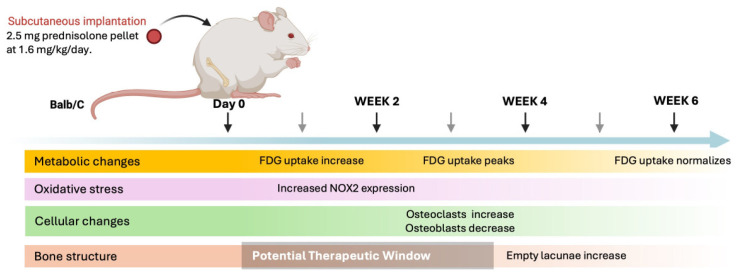
Schematic timeline illustrating the sequence of biological and imaging events observed after implantation of a sustained-release prednisolone pellet (1.6 mg/kg/day). Continuous corticosteroid exposure is initiated at day 0 and maintained throughout the 6-week study period. Increased [18F]-FDG uptake in the femoral head is detected by micro-PET/CT as early as 2 weeks, peaks at 4 weeks, and partially normalizes by 6 weeks, likely reflecting metabolic activity from multiple cell populations within the bone microenvironment. In parallel, oxidative stress is evidenced by increased NOX2 immunoreactivity beginning at 2 weeks and persisting through later time points. These early metabolic and redox changes coincide with increased osteoclast activity, as indicated by TRAP-positive cell accumulation, and suppression of osteoblast activity, assessed by reduced ALP staining along trabecular bone surfaces. Histological evidence of osteonecrosis, defined by the appearance of empty osteocyte lacunae within trabecular bone, becomes apparent by 4 weeks and increases by 6 weeks, confirming successful ONFH induction. The shaded region highlights a proposed early therapeutic window during which metabolic and oxidative alterations precede overt structural bone damage.

We have established and validated a murine model of corticosteroid-associated ONFH using a subcutaneous pellet with sustained release of a physiologically relevant dosage of prednisolone. At 3 weeks, ^18^F-FDG uptake in the femoral head was significantly higher in the prednisolone group compared to sham pellet controls, indicative of mitochondrial dysfunction and oxidative stress. At 4 weeks, femora exposed to prednisolone showed increased numbers of empty lacunae (the hallmark of ONFH), increased oxidative stress, increased osteoclastogenesis and decreased osteoblast activity, compared to sham pellets. These findings suggest that excessive oxidative stress may be a potential early marker for the development of corticosteroid-associated ONFH and for decisions for timely intervention to mitigate these adverse findings (Figure 7).

## Figures and Tables

**Table 1:** Bone mineral density.

	BMD 4 Weeks (mg/cm^2^)		BMD 6 Weeks (mg/cm^2^)	
	Whole body	Spine	Whole Body	Spine
SHAM	83.7 ± 1.4	92.2 ± 2.6	84.3 ± 3.9	96.4 ± 5.4
PRED	82.8 ± 1.0	89.2 ± 3.0	80.9 ± 2.5	87.4 ± 1.7

## Data Availability

The data presented in this study is available on request from the corresponding author. The data is not publicly available due to confidentiality concerns.

## Supplementary Materials

The following supporting information can be downloaded at https://www.mdpi.com/article/10.3390/bioengineering13010116/s1, Figure S1: Representative images illustrating the ImageJ workflow; high-resolution histology images.

## References

[1] Velentza L., Zaman F., Savendahl L. (2021). Bone health in glucocorticoid-treated childhood acute lymphoblastic leukemia. Crit. Rev. Oncol. Hematol..

[2] Shinohara I., Inui A., Hwang K., Murayama M., Susuki Y., Uno T., Gao Q., Morita M., Chow S.K., Tsubosaka M. (2025). Leveraging AI models for lesion detection in osteonecrosis of the femoral head and T1-weighted MRI generation from radiographs. J. Orthop. Res..

[3] Sharma S., Ghosh R., Marianesan A.B., Hussain S., Pandey J.D., Kumar M. (2025). Nanostructured lipid carriers in Rheumatoid Arthritis: Treatment, advancements and applications. Inflammopharmacology.

[4] Pui C.H., Evans W.E. (2006). Treatment of acute lymphoblastic leukemia. N. Engl. J. Med..

[5] Ward L.M., Ma J., Lang B., Ho J., Alos N., Matzinger M.A., Shenouda N., Lentle B., Jaremko J.L., Wilson B. (2018). Bone Morbidity and Recovery in Children with Acute Lymphoblastic Leukemia: Results of a Six-Year Prospective Cohort Study. J. Bone Miner. Res..

[6] Bruzzi P., Predieri B., Corrias A., Marsciani A., Street M.E., Rossidivita A., Paolucci P., Iughetti L. (2014). Final height and body mass index in adult survivors of childhood acute lymphoblastic leukemia treated without cranial radiotherapy: A retrospective longitudinal multicenter Italian study. BMC Pediatr..

[7] den Hoed M.A., Klap B.C., te Winkel M.L., Pieters R., van Waas M., Neggers S.J., Boot A.M., Blijdorp K., van Dorp W., Pluijm S.M. (2015). Bone mineral density after childhood cancer in 346 long-term adult survivors of childhood cancer. Osteoporos. Int..

[8] Kunstreich M., Kummer S., Laws H.J., Borkhardt A., Kuhlen M. (2016). Osteonecrosis in children with acute lymphoblastic leukemia. Haematologica.

[9] Girard P., Auquier P., Barlogis V., Contet A., Poiree M., Demeocq F., Berbis J., Herrmann I., Villes V., Sirvent N. (2013). Symptomatic osteonecrosis in childhood leukemia survivors: Prevalence, risk factors and impact on quality of life in adulthood. Haematologica.

[10] Arico M., Boccalatte M.F., Silvestri D., Barisone E., Messina C., Chiesa R., Santoro N., Tamaro P., Lippi A., Gallisai D. (2003). Osteonecrosis: An emerging complication of intensive chemotherapy for childhood acute lymphoblastic leukemia. Haematologica.

[11] Pujol O., Aguirre M., Gargallo A., Uria M.L., Riera L., Pacha D. (2021). Pulmonary Embolism after Core Decompression of the Femoral Head Using Injectable Bone Graft Substitute: A Case Report. Hip Pelvis.

[12] Xu H., Zeng Q., Zou K., Huang H., Chen J., Wang P., Yuan W., Xiao L., Tong P., Jin H. (2023). Glucocorticoid-induced activation of NOX/ROS/NF-kappaB signaling in MSCs contributes to the development of GONFH. Apoptosis.

[13] Chen K., Liu Y., He J., Pavlos N., Wang C., Kenny J., Yuan J., Zhang Q., Xu J., He W. (2020). Steroid-induced osteonecrosis of the femoral head reveals enhanced reactive oxygen species and hyperactive osteoclasts. Int. J. Biol. Sci..

[14] Yang N., Sun H., Xue Y., Zhang W., Wang H., Tao H., Liang X., Li M., Xu Y., Chen L. (2021). Inhibition of MAGL activates the Keap1/Nrf2 pathway to attenuate glucocorticoid-induced osteonecrosis of the femoral head. Clin. Transl. Med..

[15] Wu X., Sun W., Tan M. (2019). Noncoding RNAs in Steroid-Induced Osteonecrosis of the Femoral Head. Biomed. Res. Int..

[16] Lu B.B., Li K.H. (2012). Lipoic acid prevents steroid-induced osteonecrosis in rabbits. Rheumatol. Int..

[17] Zhang X., You J.M., Dong X.J., Wu Y. (2020). Administration of mircoRNA-135b-reinforced exosomes derived from MSCs ameliorates glucocorticoid-induced osteonecrosis of femoral head (ONFH) in rats. J. Cell. Mol. Med..

[18] Maruyama M., Lin T., Kaminow N.I., Thio T., Storaci H.W., Pan C.C., Yao Z., Takagi M., Goodman S.B., Yang Y.P. (2021). The efficacy of core decompression for steroid-associated osteonecrosis of the femoral head in rabbits. J. Orthop. Res..

[19] Chen C., Fu L., Luo Y., Zeng W., Qi X., Wei Y., Chen L., Zhao X., Li D., Tian M. (2023). Engineered Exosome-Functionalized Extracellular Matrix-Mimicking Hydrogel for Promoting Bone Repair in Glucocorticoid-Induced Osteonecrosis of the Femoral Head. ACS Appl. Mater. Interfaces.

[20] Sivapackiam J., Liao F., Zhou D., Shoghi K.I., Gropler R.J., Gelman A.E., Sharma V. (2020). Galuminox: Preclinical validation of a novel PET tracer for non-invasive imaging of oxidative stress in vivo. Redox Biol..

[21] Shabsigh M., Solomon L.A. (2024). Peptide PET Imaging: A Review of Recent Developments and a Look at the Future of Radiometal-Labeled Peptides in Medicine. Chem. Biomed. Imaging.

[22] Loft M., Clemmensen A., Christensen E.N., Denholt C.L., Johannesen H.H., Gillings N., Carlsen E.A., Clausen M.M., Hutchings M., Andersen T.L. (2025). First-in-Human: Simultaneous Hyperpolarized 1-13 C-Pyruvate Magnetic Resonance Spectroscopy and 18 F-FDG PET (hyperPET) Imaging of a Patient with Lymphoma. Clin. Nucl. Med..

[23] Ozturk A.E., Sahin R., Ergul N., Cermik T.F., Arslan E. (2024). A Comparison of 18 F-FDG PET/CT and 68 Ga-PSMA PET/CT in Detecting Osteonecrosis of the Jaw in a Patient with Prostate Cancer. Clin. Nucl. Med..

[24] Watanabe S., Nakajima K., Kinuya S. (2019). ^☆^Symposium: Imaging modalities for drug-related osteonecrosis of the jaw (5), utility of bone scintigraphy and ^18^F-FDG PET/CT in early detection and risk assessment of medication-related osteonecrosis of the jaw (secondary publication). Jpn. Dent. Sci. Rev..

[25] Thiele S., Baschant U., Rauch A., Rauner M. (2014). Instructions for producing a mouse model of glucocorticoid-induced osteoporosis. Bonekey Rep..

[26] Gerard C., Gallez A., Dubois C., Drion P., Delahaut P., Quertemont E., Noel A., Pequeux C. (2017). Accurate Control of 17beta-Estradiol Long-Term Release Increases Reliability and Reproducibility of Preclinical Animal Studies. J. Mammary Gland. Biol. Neoplasia.

[27] Gustafsson K.L., Farman H.H., Nilsson K.H., Henning P., Moverare-Skrtic S., Lionikaite V., Lawenius L., Engdahl C., Ohlsson C., Lagerquist M.K. (2021). Arginine site 264 in murine estrogen receptor-alpha is dispensable for the regulation of the skeleton. Am. J. Physiol. Endocrinol. Metab..

[28] Shinohara I., Inui A., Murayama M., Susuki Y., Gao Q., Chow S.K., Mifune Y., Matsumoto T., Kuroda R., Goodman S.B. (2024). Quantification of Empty Lacunae in Tissue Sections of Osteonecrosis of the Femoral Head Using YOLOv8 Artificial Intelligence Model. J. Biomed. Mater. Res. B Appl. Biomater..

[29] Toya M., Kushioka J., Shen H., Utsunomiya T., Hirata H., Tsubosaka M., Gao Q., Chow S.K., Zhang N., Goodman S.B. (2024). Sex differences of NF-kappaB-targeted therapy for mitigating osteoporosis associated with chronic inflammation of bone. Bone Jt. Res..

[30] Utsunomiya T., Zhang N., Lin T., Kohno Y., Ueno M., Maruyama M., Huang E., Rhee C., Yao Z., Goodman S.B. (2021). Suppression of NF-kappaB-induced chronic inflammation mitigates inflammatory osteolysis in the murine continuous polyethylene particle infusion model. J. Biomed. Mater. Res. Part A.

[31] Fenton C.G., Doig C.L., Fareed S., Naylor A., Morrell A.P., Addison O., Wehmeyer C., Buckley C.D., Cooper M.S., Lavery G.G. (2019). 11beta-HSD1 plays a critical role in trabecular bone loss associated with systemic glucocorticoid therapy. Arthritis Res. Ther..

[32] Wang Y., Guo Q., Hei H., Tao J., Zhou Y., Dong J., Xin H., Cai H., Gao J., Yu K. (2019). BK ablation attenuates osteoblast bone formation via integrin pathway. Cell Death Dis..

[33] Shinohara I., Mifune Y., Inui A., Nishimoto H., Yamaura K., Mukohara S., Yoshikawa T., Kato T., Furukawa T., Hoshino Y. (2022). Biochemical Markers of Aging (Advanced Glycation End Products) and Degeneration Are Increased in Type 3 Rotator Cuff Tendon Stumps with Increased Signal Intensity Changes on MRI. Am. J. Sports Med..

[34] Tsubosaka M., Maruyama M., Lui E., Kushioka J., Toya M., Gao Q., Shen H., Li X., Chow S.K., Zhang N. (2024). Preclinical models for studying corticosteroid-induced osteonecrosis of the femoral head. J. Biomed. Mater. Res. B Appl. Biomater..

[35] Tsubosaka M., Maruyama M., Lui E., Moeinzadeh S., Huang E.E., Kushioka J., Hirata H., Jain C., Storaci H.W., Chan C. (2023). The efficiency of genetically modified mesenchymal stromal cells combined with a functionally graded scaffold for bone regeneration in corticosteroid-induced osteonecrosis of the femoral head in rabbits. J. Biomed. Mater. Res. Part A.

[36] Mont M.A., Salem H.S., Piuzzi N.S., Goodman S.B., Jones L.C. (2020). Nontraumatic Osteonecrosis of the Femoral Head: Where Do We Stand Today? A 5-Year Update. J. Bone Jt. Surg. Am..

[37] Ichiseki T., Matsumoto T., Nishino M., Kaneuji A., Katsuda S. (2004). Oxidative stress and vascular permeability in steroid-induced osteonecrosis model. J. Orthop. Sci..

[38] Sheng H., Sheng C.J., Cheng X.Y., Zhang G., Lee K.M., Leung K.S., Qu S., Qin L. (2013). Pathomorphological changes of bone marrow adipocytes in process of steroid-associated osteonecrosis. Int. J. Clin. Exp. Pathol..

[39] Shinohara I., Tsubosaka M., Toya M., Lee M.L., Kushioka J., Murayama M., Gao Q., Li X., Zhang N., Chow S.K. (2023). C-C Motif Chemokine Ligand 2 Enhances Macrophage Chemotaxis, Osteogenesis, and Angiogenesis during the Inflammatory Phase of Bone Regeneration. Biomolecules.

[40] Zhang X., Feng C., Yuan T., Wang Y., Wang H., Lu Q., Lv Y., Li Z., Fu C., Sun S. (2024). Inhibition of protein disulfide isomerase mitigates steroid-induced osteonecrosis of the femoral head by suppressing osteoclast activity through the reduction of cellular oxidative stress. Chem. Biol. Interact..

[41] Glueck C.J., Freiberg R.A., Wang P. (2003). Role of thrombosis in osteonecrosis. Curr. Hematol. Rep..

[42] Ichiseki T., Kaneuji A., Ueda Y., Nakagawa S., Mikami T., Fukui K., Matsumoto T. (2011). Osteonecrosis development in a novel rat model characterized by a single application of oxidative stress. Arthritis Rheum..

[43] Yamamoto T., Irisa T., Sugioka Y., Sueishi K. (1997). Effects of pulse methylprednisolone on bone and marrow tissues: Corticosteroid-induced osteonecrosis in rabbits. Arthritis Rheum..

[44] Teitelbaum S.L. (2011). The osteoclast and its unique cytoskeleton. Ann. N. Y. Acad. Sci..

[45] Liu X., Li Q., Sheng J., Hu B., Zhu Z., Zhou S., Yin J., Gong Q., Wang Y., Zhang C. (2016). Unique plasma metabolomic signature of osteonecrosis of the femoral head. J. Orthop. Res..

[46] Cekuc M.S., Ergul Y.S., Pius A.K., Meagan M., Shinohara I., Murayama M., Susuki Y., Ma C., Morita M., Chow S.K. (2024). Metformin Modulates Cell Oxidative Stress to Mitigate Corticosteroid-Induced Suppression of Osteogenesis in a 3D Model. J. Inflamm. Res..

[47] Woods C., Wang G., Milner T.A., Glass M.J. (2024). Tumor necrosis factor alpha induces NOX2-dependent reactive oxygen species production in hypothalamic paraventricular nucleus neurons following angiotensin II infusion. Neurochem. Int..

[48] Ryu K.N., Jin W., Park J.S. (2014). Radiography, MRI, CT, Bone Scan, and PET-CT. Osteonecrosis.

[49] Cole H.A., Yuasa M., Hawley G., Cates J.M., Nyman J.S., Schoenecker J.G. (2013). Differential development of the distal and proximal femoral epiphysis and physis in mice. Bone.

[50] Kim J., Lee S.K., Kim J.Y., Kim J.H. (2023). CT and MRI findings beyond the subchondral bone in osteonecrosis of the femoral head to distinguish between ARCO stages 2 and 3A. Eur. Radiol..

[51] Ince D.C., Shah V.P., Kikuchi K., O’Connor K.P., Yanik E.L., Clohisy J.C., Pascual-Garrido C. (2025). The Femoral Head Edema Zone: A Novel Classification Scheme to Better Predict Osteonecrosis Progression. J. Arthroplast..

[52] Guntur A.R., Gerencser A.A., Le P.T., DeMambro V.E., Bornstein S.A., Mookerjee S.A., Maridas D.E., Clemmons D.E., Brand M.D., Rosen C.J. (2018). Osteoblast-like MC3T3-E1 Cells Prefer Glycolysis for ATP Production but Adipocyte-like 3T3-L1 Cells Prefer Oxidative Phosphorylation. J. Bone Miner. Res..

[53] Lee W.C., Ji X., Nissim I., Long F. (2020). Malic Enzyme Couples Mitochondria with Aerobic Glycolysis in Osteoblasts. Cell Rep..

[54] Xu X., Shen Y., Lv H., Zhao J., Li X., Gao L., Ren S., Zhang X. (2021). Tanshinone I Mitigates Steroid-Induced Osteonecrosis of the Femoral Head and Activates the Nrf2 Signaling Pathway in Rats. Evid. Based Complement. Altern. Med..

[55] Jordan E., Varady N.H., Hosseinzadeh S., Smith S., Chen A.F., Mont M., Iorio R. (2023). Femoral Head Osteonecrosis: Computed Tomography Not Needed to Identify Collapse When Using the Association Research Circulation Osseous Staging System. Arthroplast. Today.

[56] Yoon B.H., Mont M.A., Koo K.H., Chen C.H., Cheng E.Y., Cui Q., Drescher W., Gangji V., Goodman S.B., Ha Y.C. (2020). The 2019 Revised Version of Association Research Circulation Osseous Staging System of Osteonecrosis of the Femoral Head. J. Arthroplast..

[57] Koo K.H., Mont M.A., Cui Q., Hines J.T., Yoon B.H., Novicoff W.M., Lee Y.J., Cheng E.Y., Drescher W., Hernigou P. (2022). The 2021 Association Research Circulation Osseous Classification for Early-Stage Osteonecrosis of the Femoral Head to Computed Tomography-Based Study. J Arthroplast..

[58] Kawedia J.D., Kaste S.C., Pei D., Panetta J.C., Cai X., Cheng C., Neale G., Howard S.C., Evans W.E., Pui C.H. (2011). Pharmacokinetic, pharmacodynamic, and pharmacogenetic determinants of osteonecrosis in children with acute lymphoblastic leukemia. Blood.

