# Supplementary material for: A Novel Murine Model to Study the Early Biological Events of Corticosteroid-Associated Osteonecrosis of the Femoral Head
Source: Bioengineering (Basel). 2026 Jan 20;13(1):116. doi: 10.3390/bioengineering13010116 (PMC12837465; doi:10.3390/bioengineering13010116)
Supplement: Supplementary file 1 [file bioengineering-13-00116-s001.zip › bioengineering-4074501-supplementary.pdf]

Manuscript ID: bioengineering-4074501

A Novel Murine Model to Study the Early Biological Events of Corticosteroid Associated Osteonecrosis of the Femoral Head

Issei Shinohara, Yosuke Susuki, Simon Kwoon-Ho Chow*, Pierre Cheung, Abraham S Moses, Masatoshi Murayama, Mayu Morita, Tomohiro Uno, Qi Gao, Chao Ma, Takahiro Igei, Corinne Beinat*, Stuart B Goodman*

Supplementary Materials

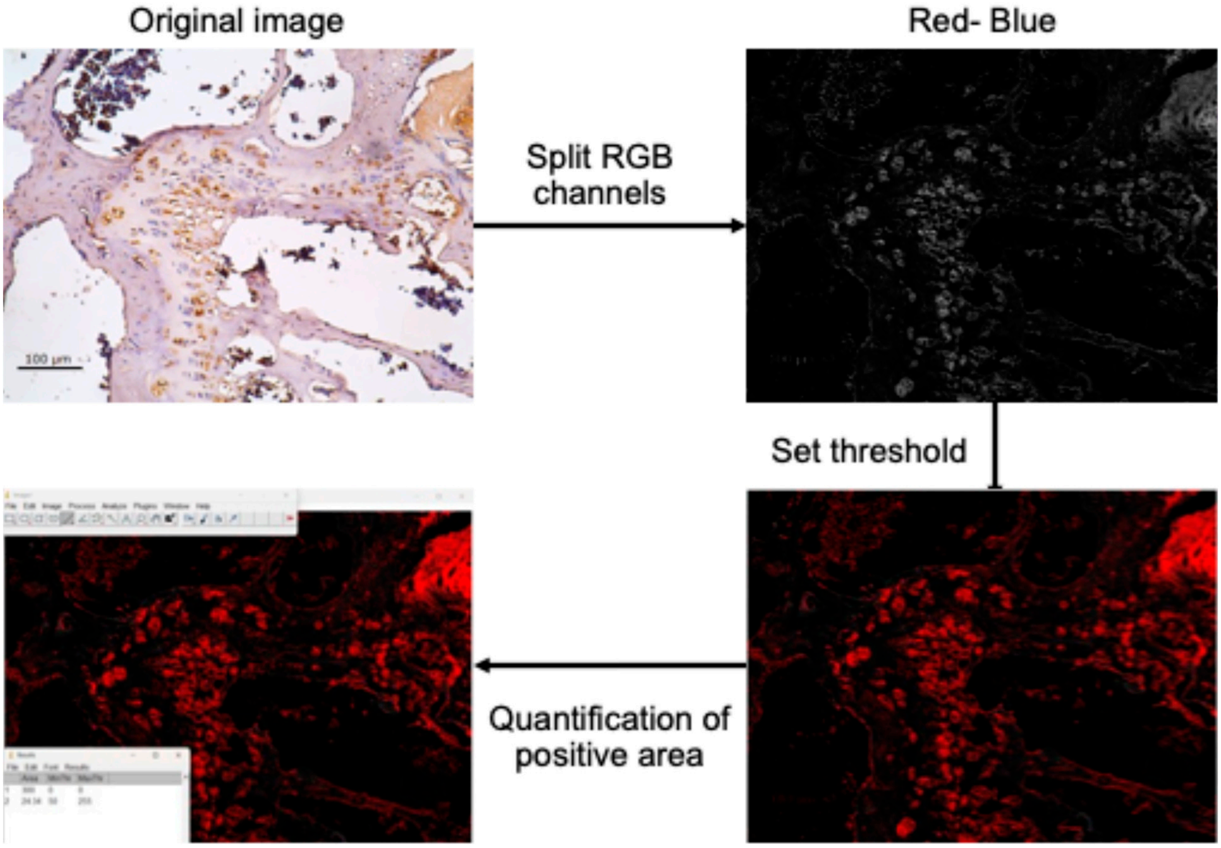

Figure S1 Representative images illustrating the ImageJ workflow used for quantification of NOX2 immunoreactivity in femoral head sections. Original brightfield images of NOX2 immunohistochemical staining (DAB, brown) were first imported into ImageJ and separated into red, green, and blue (RGB) channels. The channel providing optimal contrast between DAB-positive staining and background was selected for further analysis. Images were then subjected to fixed thresholding to segment DAB-positive signals, which were visually validated against the original image. The segmented DAB-positive area was quantified and normalized to the total bone area within the region of interest, excluding non-bone and marrow spaces. Quantification is expressed as the percentage of NOX2-positive area relative to total bone area. Identical threshold settings were applied across all samples within the same staining batch to ensure consistency.
